# Ferrocenyl­phospho­nic acid

**DOI:** 10.1107/S1600536811027206

**Published:** 2011-07-13

**Authors:** Bao-Zhang Yang, Chao Xu, Tai-Ke Duan, Qun Chen, Qian-Feng Zhang

**Affiliations:** a Institute of Molecular Engineering and Applied Chemsitry, Anhui University of Technology, Ma’anshan, Anhui 243002, People’s Republic of China; b Department of Applied Chemistry, School of Petrochemical Engineering, Changzhou University, Jiangsu 213164, People’s Republic of China

## Abstract

In the title compound, [Fe(C_5_H_5_)(C_5_H_6_O_3_P)], the phosphate group is bonded to the ferrocene unit with a P—C bond length of 1.749 (3) Å. In the crystal, six ferrocenyl­phospho­nic acid mol­ecules are connected by 12 strong inter­molecular O—H⋯O hydrogen bonds, leading to the formation of a highly distorted octa­hedral cage. The volume of the octa­hedral cage is about 270 Å^3^.

## Related literature

For background to ferrocenyl­phospho­nates and ferrocenyl derivatives, see: Alley & Henderson (2001 ▶); Henderson & Alley (2001 ▶); Oms *et al.* (2004*a* ▶,*b* ▶, 2005 ▶).
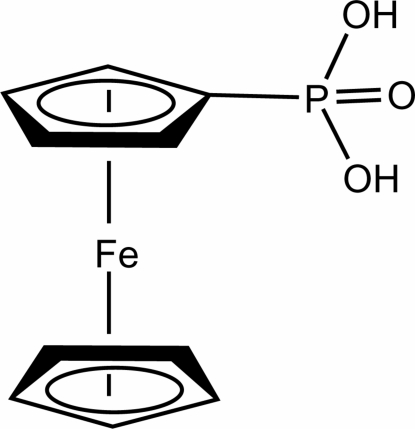


## Experimental

### 

#### Crystal data

[Fe(C_5_H_5_)(C_5_H_6_O_3_P)]
*M* *_r_* = 266.01
Trigonal, 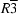

*a* = 19.7329 (9) Å
*c* = 14.7338 (4) Å
*V* = 4968.5 (5) Å^3^
*Z* = 18
Mo *K*α radiation
μ = 1.49 mm^−1^
*T* = 296 K
0.20 × 0.16 × 0.11 mm

#### Data collection

Bruker APEX CCD diffractometer
Absorption correction: multi-scan (*SADABS*; Sheldrick, 1996 ▶) *T*_min_ = 0.755, *T*_max_ = 0.853
26557 measured reflections
2500 independent reflections
1956 reflections with *I* > 2σ(*I*)
*R*_int_ = 0.044

#### Refinement

*R*[*F* ^2^ > 2σ(*F* ^2^)] = 0.040
*wR*(*F* ^2^) = 0.098
*S* = 1.03
2500 reflections
136 parameters
H-atom parameters constrained
Δρ_max_ = 0.65 e Å^−3^
Δρ_min_ = −0.51 e Å^−3^

### 

Data collection: *SMART* (Bruker, 2007 ▶); cell refinement: *SAINT* (Bruker, 2007 ▶); data reduction: *SAINT*; program(s) used to solve structure: *SHELXS97* (Sheldrick, 2008 ▶); program(s) used to refine structure: *SHELXL97* (Sheldrick, 2008 ▶); molecular graphics: *SHELXTL* (Sheldrick, 2008 ▶); software used to prepare material for publication: *SHELXTL*.

## Supplementary Material

Crystal structure: contains datablock(s) I, global. DOI: 10.1107/S1600536811027206/hy2442sup1.cif

Structure factors: contains datablock(s) I. DOI: 10.1107/S1600536811027206/hy2442Isup2.hkl

Additional supplementary materials:  crystallographic information; 3D view; checkCIF report

## Figures and Tables

**Table 1:** Hydrogen-bond geometry (Å, °)

*D*—H⋯*A*	*D*—H	H⋯*A*	*D*⋯*A*	*D*—H⋯*A*
O2—H2⋯O1^i^	0.82	1.76	2.559 (3)	165
O3—H3⋯O1^ii^	0.82	1.79	2.557 (3)	154

Symmetry codes: (i) 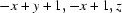; (ii) 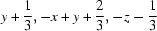.

**Fig. 1.**
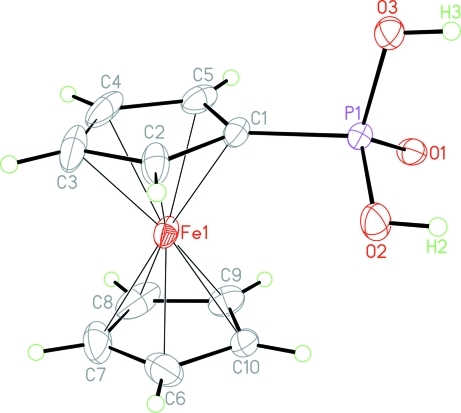
Molecular structure of the title compound, showing displacement ellipsoids at the 50% probability level.

**Fig. 2.**
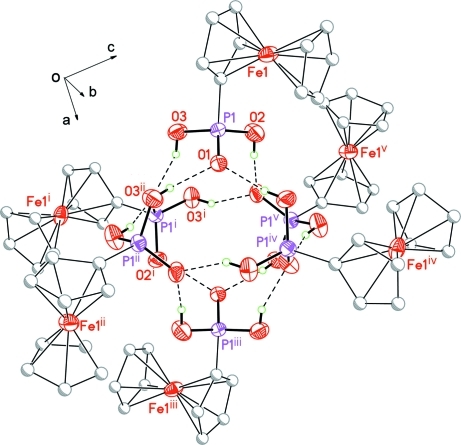
Six ferrocenylphosphonic acid molecules are connected by intermolecular O—H···O hydrogen bonds (dashed lines), forming a distorted octahedral cage. [Symmetry codes: (i) 1/3+y, 2/3-x+y, -1/3-z; (ii) 1/3+x-y, -1/3+x, -1/3-z; (iii) 3/4-x, 2/3-y, -1/3-z; (iv) 1-y, x-y, z; (v) 1-x+y, 1-x, z.]

## supplementary crystallographic information

### Comment

Ferrocene and ferrocenyl derivatives are well known for their redox activities and immobilization behaviors with metal ions (Alley & Henderson, 2001; Oms *et al.*, 2004*a*). Ferrocenylphosphonates are ideal building block candidates for incorporation with transition metal ions due to the strong coordination behavior of P═O groups and the high stability of the formed P—O—M (M = metal ion) bonds (Henderson & Alley, 2001; Oms *et al.*, 2005). However, only a few metal ferrocenylphosphonate compounds have been reported from related literature, probably due to the low yield of ferrocenylphosphonic acid in its synthesis (Oms *et al.*, 2004*b*). In this paper, we reported the preparation of the crystalline ferrocenylphosphonic acid in a relatively high yield and its crystal structure.

The molecular structure of the title compound is depicted in Fig. 1. The Fe atom lies between two cyclopentadiene (Cp) planes, with an average Fe—Cp(centroid) of 1.649 (2) Å. The [PO(OH)_2_] group is bonded to the ferrocene molecule *via* a P—C bond with a bond length of 1.749 (3) Å. The average bond length of P—O [1.543 (2) Å] is obviously longer than that of P═O [1.498 (2) Å]. The P atom is located in a slightly distorted tetrahedral environment, with the O—P—O and C—P—O bond angles in the ranges of 110.01 (13)–112.81 (13)° and 104.65 (13)—112.69 (13)°, respectively. The bond lengths and angles in the title compound are similar to those found in [FcCH_2_P(O)(OH)_2_] [Fc = (η^5^-C_5_H_4_)Fe(η^5^-C_5_H_5_)] (Oms *et al.*, 2004*b*). It is interesting to note that six ferrocenylphosphonic acid molecules are connected by twelve strong intermolecular O—H···O hydrogen bonds (Table 1), leading to the formation of a highly distorted octahedral cage, as shown in Fig. 2. The volume of the cavity of the octahedral cage is about 270 Å^3^. The crystal packing is stabilized by these intermolecular hydrogen-bonding interactions.

### Experimental

Ferrocenylphosphonic acid was prepared according to literature (Oms *et al.*, 2004*b*). All synthesis was taken in oven-dried glassware under a nitrogen atmosphere using standard Schlenk techniques. Me_3_SiBr (4.5 g, 30 mmol) was dropwise added to a solution of diethyl ferrocenylphosphonate (3.18 g, 10 mmol) in 20 ml CH_2_Cl_2_ at room temperature. After the mixture was stirred for 12 h, the solvent was removed under low pressure and the oil residues was dissolved in 20 ml MeCN and then treated with 5 ml H_2_O to precipitate the title compound. The precipitate was collected and washed with CH_2_Cl_2_ and Et_2_O (yield: 2.05 g, 80%). Single crystals suitable for X-ray analysis were obtained by recrystallization from methanol/Et_2_O. Analysis, calculated, for C_10_H_11_FeO_3_P: C 45.1, H 4.16%; found: C 45.0, H 4.04%.

### Refinement

H atoms were positioned geometrically and refined using a riding model, with C—H = 0.93 and O—H = 0.82 Å and with *U*_iso_(H) = 1.2(1.5 for hydroxyl)*U*_eq_(C,O).

### Crystal data

**Table:**

[Fe(C_5_H_5_)(C_5_H_6_O_3_P)]	*D*_x_ = 1.600 Mg m^−^^3^
*M**_r_* = 266.01	Mo *K*α radiation, λ = 0.71073 Å
Trigonal, *R*3	Cell parameters from 5069 reflections
Hall symbol: -R 3	θ = 2.8–22.9°
*a* = 19.7329 (9) Å	µ = 1.49 mm^−^^1^
*c* = 14.7338 (4) Å	*T* = 296 K
*V* = 4968.5 (5) Å^3^	Block, red
*Z* = 18	0.20 × 0.16 × 0.11 mm
*F*(000) = 2448	

### Data collection

**Table:**

Bruker APEX CCD diffractometer	2500 independent reflections
Radiation source: fine-focus sealed tube	1956 reflections with *I* > 2σ(*I*)
graphite	*R*_int_ = 0.044
φ and ω scans	θ_max_ = 27.5°, θ_min_ = 3.0°
Absorption correction: multi-scan (*SADABS*; Sheldrick, 1996)	*h* = −25→25
*T*_min_ = 0.755, *T*_max_ = 0.853	*k* = −25→25
26557 measured reflections	*l* = −19→19

### Refinement

**Table:**

Refinement on *F*^2^	Primary atom site location: structure-invariant direct methods
Least-squares matrix: full	Secondary atom site location: difference Fourier map
*R*[*F*^2^ > 2σ(*F*^2^)] = 0.040	Hydrogen site location: inferred from neighbouring sites
*wR*(*F*^2^) = 0.098	H-atom parameters constrained
*S* = 1.03	*w* = 1/[σ^2^(*F*_o_^2^) + (0.041*P*)^2^ + 9.0107*P*] where *P* = (*F*_o_^2^ + 2*F*_c_^2^)/3
2500 reflections	(Δ/σ)_max_ = 0.001
136 parameters	Δρ_max_ = 0.65 e Å^−^^3^
0 restraints	Δρ_min_ = −0.51 e Å^−^^3^

### Fractional atomic coordinates and isotropic or equivalent isotropic displacement parameters (Å^2^)

**Table:**

	*x*	*y*	*z*	*U*_iso_*/*U*_eq_	
Fe1	0.62280 (2)	0.51797 (2)	0.15180 (3)	0.05726 (16)	
P1	0.69358 (4)	0.48371 (4)	−0.03885 (4)	0.04430 (18)	
O1	0.61726 (12)	0.41531 (10)	−0.06806 (12)	0.0603 (5)	
O2	0.74425 (14)	0.45939 (14)	0.01591 (14)	0.0750 (7)	
H2	0.7540	0.4309	−0.0152	0.113*	
O3	0.73972 (13)	0.53301 (11)	−0.12206 (14)	0.0659 (6)	
H3	0.7485	0.5058	−0.1567	0.099*	
C1	0.68160 (15)	0.54767 (14)	0.03269 (19)	0.0503 (6)	
C2	0.73203 (19)	0.59400 (17)	0.1052 (2)	0.0733 (9)	
H2A	0.7784	0.5961	0.1224	0.088*	
C3	0.6995 (3)	0.6354 (2)	0.1457 (3)	0.1043 (17)	
H3A	0.7200	0.6690	0.1952	0.125*	
C4	0.6307 (3)	0.6176 (2)	0.0989 (3)	0.1058 (17)	
H4	0.5984	0.6381	0.1118	0.127*	
C5	0.6182 (2)	0.5633 (2)	0.0289 (3)	0.0745 (9)	
H5	0.5766	0.5418	−0.0118	0.089*	
C6	0.6355 (3)	0.4572 (3)	0.2550 (3)	0.0955 (12)	
H6	0.6831	0.4657	0.2776	0.115*	
C7	0.5957 (4)	0.4929 (3)	0.2849 (3)	0.121 (2)	
H7	0.6112	0.5296	0.3314	0.145*	
C8	0.5269 (3)	0.4645 (3)	0.2330 (4)	0.120 (2)	
H8	0.4888	0.4788	0.2387	0.144*	
C9	0.5268 (2)	0.4100 (2)	0.1706 (3)	0.0803 (10)	
H9	0.4887	0.3818	0.1273	0.096*	
C10	0.5939 (2)	0.40671 (18)	0.1861 (2)	0.0677 (8)	
H10	0.6086	0.3752	0.1548	0.081*	

### Atomic displacement parameters (Å^2^)

**Table:**

	*U*^11^	*U*^22^	*U*^33^	*U*^12^	*U*^13^	*U*^23^
Fe1	0.0606 (3)	0.0423 (2)	0.0670 (3)	0.02441 (19)	0.0177 (2)	−0.00354 (18)
P1	0.0532 (4)	0.0378 (3)	0.0442 (4)	0.0245 (3)	−0.0017 (3)	−0.0026 (3)
O1	0.0731 (13)	0.0404 (10)	0.0487 (10)	0.0143 (9)	−0.0092 (9)	−0.0005 (8)
O2	0.1037 (17)	0.1009 (17)	0.0606 (12)	0.0813 (15)	−0.0152 (12)	−0.0171 (12)
O3	0.0780 (14)	0.0469 (11)	0.0628 (12)	0.0238 (10)	0.0178 (10)	0.0001 (9)
C1	0.0497 (14)	0.0372 (13)	0.0620 (16)	0.0202 (11)	0.0101 (12)	0.0008 (11)
C2	0.0607 (18)	0.0508 (17)	0.081 (2)	0.0074 (14)	0.0152 (16)	−0.0202 (15)
C3	0.118 (3)	0.0473 (19)	0.119 (3)	0.020 (2)	0.057 (3)	−0.019 (2)
C4	0.138 (4)	0.061 (2)	0.146 (4)	0.070 (3)	0.084 (3)	0.035 (2)
C5	0.079 (2)	0.068 (2)	0.096 (2)	0.0522 (18)	0.0258 (19)	0.0260 (18)
C6	0.107 (3)	0.098 (3)	0.065 (2)	0.039 (3)	−0.011 (2)	0.005 (2)
C7	0.180 (6)	0.080 (3)	0.076 (3)	0.045 (3)	0.052 (3)	−0.007 (2)
C8	0.120 (4)	0.098 (3)	0.173 (5)	0.076 (3)	0.103 (4)	0.068 (3)
C9	0.060 (2)	0.0574 (19)	0.103 (3)	0.0144 (16)	0.0086 (18)	0.0191 (19)
C10	0.092 (2)	0.0541 (17)	0.0598 (18)	0.0391 (17)	0.0121 (17)	0.0068 (14)

### Geometric parameters (Å, °)

**Table:**

Fe1—C1	2.022 (3)	C2—C3	1.400 (5)
Fe1—C7	2.028 (4)	C2—H2A	0.9300
Fe1—C6	2.028 (4)	C3—C4	1.401 (7)
Fe1—C8	2.032 (4)	C3—H3A	0.9300
Fe1—C2	2.033 (3)	C4—C5	1.417 (6)
Fe1—C10	2.037 (3)	C4—H4	0.9300
Fe1—C3	2.040 (4)	C5—H5	0.9300
Fe1—C9	2.041 (3)	C6—C7	1.366 (7)
Fe1—C5	2.042 (4)	C6—C10	1.371 (5)
Fe1—C4	2.046 (4)	C6—H6	0.9300
P1—O1	1.4975 (19)	C7—C8	1.407 (7)
P1—O2	1.537 (2)	C7—H7	0.9300
P1—O3	1.547 (2)	C8—C9	1.414 (6)
P1—C1	1.749 (3)	C8—H8	0.9300
O2—H2	0.8200	C9—C10	1.377 (5)
O3—H3	0.8200	C9—H9	0.9300
C1—C5	1.432 (4)	C10—H10	0.9300
C1—C2	1.435 (4)		
			
C1—Fe1—C7	162.4 (2)	C2—C1—Fe1	69.69 (17)
C1—Fe1—C6	126.80 (16)	P1—C1—Fe1	125.37 (14)
C7—Fe1—C6	39.4 (2)	C3—C2—C1	108.4 (4)
C1—Fe1—C8	155.8 (2)	C3—C2—Fe1	70.2 (2)
C7—Fe1—C8	40.5 (2)	C1—C2—Fe1	68.87 (16)
C6—Fe1—C8	67.0 (2)	C3—C2—H2A	125.8
C1—Fe1—C2	41.44 (12)	C1—C2—H2A	125.8
C7—Fe1—C2	124.0 (2)	Fe1—C2—H2A	126.7
C6—Fe1—C2	106.88 (18)	C2—C3—C4	108.2 (4)
C8—Fe1—C2	161.9 (2)	C2—C3—Fe1	69.62 (18)
C1—Fe1—C10	109.79 (11)	C4—C3—Fe1	70.2 (2)
C7—Fe1—C10	66.42 (16)	C2—C3—H3A	125.9
C6—Fe1—C10	39.41 (15)	C4—C3—H3A	125.9
C8—Fe1—C10	66.91 (15)	Fe1—C3—H3A	125.9
C2—Fe1—C10	120.09 (15)	C3—C4—C5	109.3 (3)
C1—Fe1—C3	68.93 (13)	C3—C4—Fe1	69.7 (2)
C7—Fe1—C3	105.94 (19)	C5—C4—Fe1	69.55 (18)
C6—Fe1—C3	117.6 (2)	C3—C4—H4	125.4
C8—Fe1—C3	125.81 (19)	C5—C4—H4	125.4
C2—Fe1—C3	40.21 (14)	Fe1—C4—H4	126.9
C10—Fe1—C3	152.2 (2)	C4—C5—C1	107.0 (4)
C1—Fe1—C9	121.25 (14)	C4—C5—Fe1	69.9 (2)
C7—Fe1—C9	67.7 (2)	C1—C5—Fe1	68.65 (17)
C6—Fe1—C9	66.79 (17)	C4—C5—H5	126.5
C8—Fe1—C9	40.61 (19)	C1—C5—H5	126.5
C2—Fe1—C9	154.70 (15)	Fe1—C5—H5	126.5
C10—Fe1—C9	39.46 (15)	C7—C6—C10	108.9 (4)
C3—Fe1—C9	164.92 (17)	C7—C6—Fe1	70.3 (3)
C1—Fe1—C5	41.25 (12)	C10—C6—Fe1	70.6 (2)
C7—Fe1—C5	154.2 (2)	C7—C6—H6	125.6
C6—Fe1—C5	165.83 (17)	C10—C6—H6	125.6
C8—Fe1—C5	121.1 (2)	Fe1—C6—H6	125.1
C2—Fe1—C5	68.93 (15)	C6—C7—C8	107.9 (4)
C10—Fe1—C5	129.96 (14)	C6—C7—Fe1	70.3 (2)
C3—Fe1—C5	68.53 (19)	C8—C7—Fe1	69.9 (3)
C9—Fe1—C5	110.85 (16)	C6—C7—H7	126.0
C1—Fe1—C4	68.53 (12)	C8—C7—H7	126.0
C7—Fe1—C4	119.1 (2)	Fe1—C7—H7	125.3
C6—Fe1—C4	151.7 (2)	C7—C8—C9	106.9 (4)
C8—Fe1—C4	109.11 (17)	C7—C8—Fe1	69.6 (2)
C2—Fe1—C4	67.60 (18)	C9—C8—Fe1	70.0 (2)
C10—Fe1—C4	167.3 (2)	C7—C8—H8	126.5
C3—Fe1—C4	40.11 (19)	C9—C8—H8	126.5
C9—Fe1—C4	129.7 (2)	Fe1—C8—H8	125.5
C5—Fe1—C4	40.57 (17)	C10—C9—C8	107.0 (4)
O1—P1—O2	112.81 (13)	C10—C9—Fe1	70.09 (19)
O1—P1—O3	110.46 (11)	C8—C9—Fe1	69.4 (2)
O2—P1—O3	110.01 (13)	C10—C9—H9	126.5
O1—P1—C1	112.69 (13)	C8—C9—H9	126.5
O2—P1—C1	104.65 (13)	Fe1—C9—H9	125.6
O3—P1—C1	105.86 (12)	C6—C10—C9	109.2 (4)
P1—O2—H2	109.5	C6—C10—Fe1	70.0 (2)
P1—O3—H3	109.5	C9—C10—Fe1	70.44 (19)
C5—C1—C2	107.1 (3)	C6—C10—H10	125.4
C5—C1—P1	125.4 (2)	C9—C10—H10	125.4
C2—C1—P1	127.4 (2)	Fe1—C10—H10	125.8
C5—C1—Fe1	70.10 (17)		

### Hydrogen-bond geometry (Å, °)

**Table:**

*D*—H···*A*	*D*—H	H···*A*	*D*···*A*	*D*—H···*A*
O2—H2···O1^i^	0.82	1.76	2.559 (3)	165
O3—H3···O1^ii^	0.82	1.79	2.557 (3)	154

Symmetry codes: (i) −*x*+*y*+1, −*x*+1, *z*; (ii) *y*+1/3, −*x*+*y*+2/3, −*z*−1/3.
